# Characterization and outcomes of cats with cardiomyopathy undergoing subcutaneous ureteral bypass device placement

**DOI:** 10.1093/jvimsj/aalag127

**Published:** 2026-07-01

**Authors:** Claire Cassou, Christina Plante, Catherine Vachon, Gregor Boot, Tristan Juette, Marilyn Dunn

**Affiliations:** Department of Clinical Sciences, Ontario Veterinary College, University of Guelph, Guelph, Ontario, Canada; Department of Clinical Sciences, Faculty of Veterinary Medicine, University of Montreal, Saint-Hyacinthe, Quebec, Canada; Department of Clinical Sciences, Faculty of Veterinary Medicine, University of Montreal, Saint-Hyacinthe, Quebec, Canada; Department of Clinical Sciences, Veterinary Teaching Hospital, Louisiana State University, Bâton Rouge, LA, United States; Faculty of Veterinary Medicine, University of Montreal, Saint-Hyacinthe, Quebec, Canada; Department of Clinical Sciences, Faculty of Veterinary Medicine, University of Montreal, Saint-Hyacinthe, Quebec, Canada

**Keywords:** azotemia, cardiac disease, fluid overload, survival, ureteral obstruction

## Abstract

**Background:**

Limited data exist on the characterization and outcomes of cats with cardiomyopathy and benign ureteral obstruction treated by subcutaneous ureteral bypass (SUB) placement.

**Hypothesis/Objectives:**

Characterize cardiomyopathies, assess perioperative and long-term outcomes, and determine if an association exists between left atrial size and fluid overload (FO) or survival.

**Animals:**

Ninety-four cats presented for SUB placement between 2010 and 2022 at the Veterinary Teaching Hospital of the University of Montreal.

**Methods:**

Retrospective review of medical records including physical examination, serum creatinine concentration, echocardiography, and outcome. Cats were grouped as: cardiomyopathy present (CM+) or absent (CM−).

**Results:**

Echocardiography was performed in 64/94 (68%) cats. Cardiomyopathy was diagnosed in 26/64 (40%) cats, including 85% (22/26) hypertrophic or hypertrophic obstructive cardiomyopathy. The majority of cats (22/26) had mild cardiomyopathy, with normal left atrial size (left atrial-to-aortic root diameter ratio in right parasternal short axis [LA:Ao_SAX_] < 1.6). No significant difference in duration of hospitalization, survival time, renal recovery, or frequency of FO was found between CM+ and CM− cats. Fluid overload occurred in 45% (29/64) of the cats, and was associated with shorter survival (median [IQR]: 430 [40; 1260] days vs 1020 [494; 1668] days; *P* = .005). Admission serum creatinine concentration was higher in cats with FO (mean ± SD: 11.12 ± 5.93 mg/dL vs 6.16 ± 5.44 mg/dL; *P* < .001) and was associated with worse prognosis (*P* = .01). The LA:Ao_SAX_ was not associated with FO (*P* = .26) or survival (*P* = .39).

**Conclusions and clinical importance:**

Mild cardiomyopathy was a common comorbidity but did not affect outcome and should not be considered a contraindication for SUB placement. However, the impact of more advanced cardiac disease remains unknown. In contrast, FO and higher serum creatinine concentration at admission predicted shorter survival.

## Introduction

Benign ureteral obstruction in cats commonly results from ureterolithiasis (of which 98% are composed of calcium oxalate), strictures, and exudate (pyonephrosis).^1–4^ Cats with severe obstruction may present with oliguria or anuria, are at risk of fluid overload (FO) and may have cardiovascular instability.^5–8^ Placement of a subcutaneous ureteral bypass (SUB) has become a common treatment for benign ureteral obstruction and is consistently associated with favorable outcomes.^1^^,^^9–12^

Among cats referred for benign ureteral obstruction management, 48%-54% have heart murmurs detected on presentation.^11–13^ The cause of murmurs in cats includes high cardiac output states (eg, anemia, fever), structural cardiac disease, or physiologic mechanisms such as dynamic right ventricular outflow tract obstruction (DRVOTO).^14–17^ Importantly, murmurs are inconsistently associated with underlying cardiomyopathy, and up to 22% of cats with cardiomyopathy may have no detectable murmur or gallop on auscultation.^18^^,^^19^ Despite often being asymptomatic for extended periods, cardiomyopathy can lead to life-threatening complications, including congestive heart failure (CHF), arterial thromboembolism, and is considered a risk factor for FO.^6^^,^^20^^,^^21^

Fluid overload is a widely used yet inconsistently defined term in both human and veterinary medical literature, generally referring to the pathologic accumulation of excess body fluid within the interstitial spaces resulting in hypervolemia, edema, or both. Clinically, FO often is identified by some combination of clinical signs, excessive weight gain, and markedly positive fluid balance, typically in the context of IV fluid therapy.^22–24^ It usually occurs secondary to intensive fluid administration in the context of oliguric or anuric acute kidney injury, sepsis, underlying cardiac disease, or cardiorenal syndrome, and usually reflects impaired water excretion, abnormal interstitial compartment function or both, rather than the rate of fluid administration alone.^6^^,^^7^^,^^22^ Severe anemia also can contribute to FO, as emphasized by a recent case series describing presumed high-output cardiac failure in cats without clinically relevant structural heart disease.^25^ In human medicine, FO has been widely recognized as an independent predictor of morbidity and mortality across various hospitalized populations.^26–29^ Similarly, in veterinary medicine, FO is associated with exacerbation of kidney dysfunction, increased risk of multi-organ complications, and worse outcomes in cats with renal disease, including those undergoing SUB placement, emphasizing the importance of its recognition and prevention.^11^^,^^30–32^

Although the interplay between the cardiovascular and renal systems is increasingly acknowledged in veterinary medicine, prognostic indicators in cats with cardiomyopathy and benign ureteral obstruction after SUB placement remain poorly defined. Our objectives were to characterize the types of cardiomyopathy among cats undergoing echocardiographic evaluation in the context of SUB placement and to evaluate their perioperative and long-term outcomes. Primary outcomes included whether the presence of cardiomyopathy was associated with increased risk of FO, longer hospitalization, decreased survival time, or impaired renal recovery. Secondary objectives were to identify clinical predictors of FO and to assess whether left atrial size was associated with the development of FO in this population.

## Materials and methods

### Case selection

The medical records of cats that underwent SUB placement between April 2012 and October 2022 at the Veterinary Teaching Hospital of the University of Montreal (CHUV) were retrospectively reviewed.

### Inclusion criteria and classification

Cats presented for SUB placement were included if medical records were complete and were divided into 3 groups: cats that did not undergo echocardiography (NE), cats that underwent echocardiography and were diagnosed with cardiomyopathy (CM+), and cats that underwent echocardiography but did not have cardiomyopathy (CM−). To be included in the CM+ group, echocardiography and diagnosis were required within 2 weeks before or after SUB placement. For the analysis related to FO, only echocardiographic parameters obtained before the onset of FO were included. However, echocardiographic data from all time points were considered for the remainder of the statistical analyses and diagnosis of cardiomyopathy.

Exclusion criteria included the presence of concurrent systemic arterial hypertension, hyperthyroidism, the presence of congenital cardiac anomalies, or if SUB placement was not attempted or was declined and led to euthanasia. Cats exhibiting systolic arterial hypertension upon admission, which subsequently normalized during hospitalization and did not require administration of antihypertensive medications, were included because the hypertension was deemed to be stress-induced.

### Data collection

Data collected from medical records included signalment, physical examination findings, hydration status, presence of concurrent disease, preoperative clinical signs, noninvasive blood pressure (NIBP) measurement, CBC, and serum biochemistry (including serum creatinine and total thyroxine concentration [TT4]) at admission (T_0_). Serum creatinine concentration additionally was reported at discharge (T_1_), 1 month (T_2_), 3 months (T_3_), 6 months (T_4_), and 1 year (T_5_) after SUB placement. The change in serum creatinine concentration (∆Creatinine) was defined as the difference in serum creatinine concentration between 2 results and was calculated at different time points: ∆Creatinine1 (between T_1_ and T_0_), ∆Creatinine2 (between T_2_ and T_0_), and ∆Creatinine3 (between T_3_ and T_0_). Noninvasive blood pressure measurements were obtained using either a Doppler ultrasonic flow detector or an oscillometric device (PetMAP, Ramsey Medical), depending on availability and patient compliance. Depending on the data available in the medical records, the systolic blood pressure result reported corresponded to the average of 3-5 consecutive measurements. Measurements were performed according to standardized protocols, and mean systolic blood pressure > 160 mmHg was considered indicative of systemic hypertension, in accordance with the 2018 American College of Veterinary Internal Medicine consensus guidelines on systemic hypertension in dogs and cats.^33^ Presence or absence, grade, and point of maximal intensity of heart murmur, and the presence or absence of a gallop sound or an arrhythmia on auscultation were recorded. Anemia was defined as a PCV < 28%, based on the reference intervals and clinical thresholds provided by the laboratory where the analyses were performed.

For each patient, the duration of hospitalization and outcome were recorded, as well as signs of FO and perioperative treatments administered. Signs of FO are listed in Table 1 and classified as minor or major criteria based on clinical signs of volume overload described in the current literature, as well as the signs that are routinely monitored at our institution by experienced criticalists, internists, and cardiologists.^6^^,^^23^^,^^24^^,^^34^ Fluid overload was confirmed when at least 1 minor and 1 major criterion was simultaneously present, the clinical record mentioned a decrease in fluid rate because of suspicion of FO, a diagnosis of FO was established by a board-certified cardiologist performing echocardiography, or some combination of these.

**Table 1:** Criteria for retrospective diagnosis of fluid overload in cats.

Major criteria	Minor criteria
Increased euhydrated weight ≥ 10%	Tachypnea, dyspnea
Point-of-care ultrasound	Jugular pulses
Appearance of new B-lines suggestive of pulmonary edema	Nasal discharge
Increased LA:Ao ratio	Subcutaneous effusion
Presence of pleural and/or abdominal effusion	Increased euhydrated weight (5%-10%)
Thoracic radiographs	Cardiac and pulmonary auscultation
Signs of congestion and/or pulmonary edema	Onset of a persistent gallop sound
Echocardiography	Onset or intensification of a heart murmur
Diagnosis of congestion and fluid overload by a board-certified cardiologist	Crackles or abnormal lung sounds

Abbreviation: LA:Ao ratio = left atrial-to-aortic root diameter ratio.

### Echocardiography

Echocardiography (Vivid E95, GE-Medical) was performed by a board-certified cardiologist or a board-certified internist trained in cardiology. M-mode, 2-dimensional (2D) and Doppler echocardiographic images were conventionally obtained in right and left lateral recumbency. To facilitate echocardiographic examination, sedation (gabapentin, butorphanol, buprenorphine, remifentanil, midazolam, or alfaxolone) was administered to some cats. Echocardiography reports were reviewed and the following data were extracted or, if not listed in the report, retrospectively measured from available images by a trained intern (C.C.): date of examination, echocardiographic parameters including 2D left atrial-to-aortic root diameter ratio (LA:Ao_SAX_), left ventricular wall hypertrophy, presence or absence of dynamic left or right ventricular outflow tract obstruction, and pleural or pericardial effusion. When left ventricular concentric hypertrophy was identified, the echocardiographic findings were interpreted in the context of the patient’s hydration status, additional echocardiographic parameters, and persistence of hypertrophy on subsequent evaluations to distinguish true hypertrophy from pseudohypertrophy.

Left ventricular (LV) wall thickness was measured from 2D images obtained from right parasternal long-axis LV outflow and right parasternal short-axis views. A leading-edge-to-leading-edge method of measurement was used. Left ventricular hypertrophy was defined as a maximum thickness of the interventricular septum, LV free wall segment, or both ≥ 6 mm.^35^ Hypertrophic obstructive cardiomyopathy (HOCM) was characterized by LV hypertrophy in association with systolic anterior motion of the mitral valve, and diffuse turbulence in the left ventricular outflow tract with a peak systolic velocity ≥ 2.5 m/s.^35^ Dynamic right ventricular outflow tract obstruction was identified as a turbulent systolic flow jet within the right ventricular outflow tract with a peak systolic velocity exceeding 1.7 m/s.^16^ Assessment of left atrial size was made using 2D images from the right parasternal short-axis view to calculate LA:Ao_SAX_ in early diastole, on the first diastolic frame in which aortic valve closure was visible.^36^ For each echocardiographic parameter, the average of 3 consecutive measurements was used. Classification of underlying cardiac disease (eg hypertrophic cardiomyopathy, restrictive cardiomyopathy, dilated cardiomyopathy, and unclassified cardiomyopathy) was made according to published criteria.^20^ Cardiomyopathies were classified as mild, moderate, or severe based on an arbitrary stratification of left atrium (LA) size. Cats with LA:Ao_SAX_ < 1.6, corresponding to the absence of left atrial dilatation, were classified as having mild cardiomyopathy. Moderate cardiomyopathy was defined by an LA:Ao_SAX_ between 1.6 and 1.8, consistent with mild to moderate LA enlargement, whereas severe cardiomyopathy was defined by an LA:Ao_SAX_ > 1.8, indicating marked LA dilatation.^18^^,^^36–38^

### Statistical analysis

Statistical analyses were performed using R version 4.3.1 (R Core Team, 2023).^39^ Population description (age, body weight, sex, and breed), general laboratory findings (PCV, TT4), and clinicopathologic features (blood pressure, auscultation findings) were evaluated in the 3 initial groups (NE, CM+, and CM−). However, to address our main objective, statistical analysis regarding population distribution, serum creatinine concentration, anemia, development of FO and echocardiographic parameters (LA:Ao_SAX_), focused mainly on groups CM+ and CM−. Descriptive statistics (median with IQR and mean ± SD) were reported for the relevant variables in each group. Shapiro–Wilk tests were used to assess normality of each continuous variable.

When comparing 2 groups (CM+ and CM−) for continuous variables such as age, body weight, survival time, hospitalization duration, and serum creatinine concentration at various time points (CreaT0, CreaT1, CreaT2, and CreaT3), and changes in serum creatinine concentration (ΔCreatinine1, ΔCreatinine2, and ΔCreatinine3) results were tested for normality using Shapiro–Wilk tests and for homogeneity of variances using Levene’s test. When both assumptions were met, independent *t*-tests were applied (comparison of age). If at least 1 of the 2 assumptions was not met, Mann–Whitney *U* tests were used (body weight, hospitalization duration, survival time, and serum creatinine concentration). Sex distribution (categorical variable) was compared between groups using Fisher’s exact test. For analyses involving a binary dependent variable, generalized linear models with a binomial distribution and logit link function were used. These included: effect of cardiomyopathy, anemia, number of transfusions, LA:Ao_SAX_, unilateral or bilateral SUB, serum creatinine concentration at T0 and T3, and cause of death, on the development or lack of development of FO. To test the effects of LA:Ao_SAX_ and serum creatinine concentration at T0 on survival time, linear models were used. For these models, normality of residuals was assessed using the Shapiro–Wilk test. When normality assumptions were violated, survival time was transformed. Survival time was square-root transformed in the model testing the effect of LA:Ao_SAX_ and log_10_ transformed in the model testing the effect of serum creatinine concentration at T0, because these transformations best allowed for normality of the residuals to be obtained. To identify potential discriminatory thresholds for LA:Ao_SAX_ in predicting FO, receiver operating characteristic (ROC) curves were generated. The area under the curve (AUC) and its 95% CI were estimated using bootstrap resampling with 1000 iterations. The optimal threshold was determined based on the point maximizing sensitivity and specificity. For all tests, statistical significance was set at *P* < .05.

## Results

### Study population

Between April 2012 and October 2022, 158 cats were presented to the CHUV and referred to the Interventional Medicine Service for SUB placement because of unilateral or bilateral ureteral obstruction. Placement was declined for 55 cats. Financial constraints were the sole reason in 47/55 (85%) cats, comorbidities alone accounted for 1/55 (2%) cat, and a combination of financial constraints and comorbidities was noted in 7/55 (13%) cats. Among cats with comorbidities, 3/8 had cardiomyopathy: 1 with unclassified cardiomyopathy and CHF, 1 with moderate-to-severe hypertrophic cardiomyopathy stable for 2 years before presentation (in the context of additional comorbidities including severe osteoarthritis, long-standing International Renal Interest Society [IRIS] chronic kidney disease [CKD] stage 3, and suspected inflammatory bowel disease [IBD]), and 1 with mild hypertrophic cardiomyopathy (also positive for feline immunodeficiency virus, with CKD, IBD, and chronic rhinitis). For the remaining cats euthanized because of a combination of comorbidities and financial constraints (5/7), the comorbidities included IRIS CKD stage 2 (1/7), IRIS CKD stage 3 with IBD (1/7), hyperthyroidism (1/7), hepatic lipidosis and severe pancreatitis (1/7), and suspected triaditis (1/7). In all cases, cardiac disease was not the sole reason reported for euthanasia. A total of 94 cats met the inclusion criteria and 9 cats were excluded because of persistent or previously diagnosed systemic arterial hypertension (*n* = 5), congenital heart disease (*n* = 3), and hyperthyroidism (*n* = 1).

Within 2 weeks before or after SUB placement, 64/94 cats had echocardiography performed (68%). Thirty cats did not undergo echocardiography and were assigned to the NE group. Among the 64 cats that had echocardiography performed, cardiomyopathy was diagnosed in 26/64 (40%; CM+ group), whereas 38/64 (60%) had no evidence of cardiac disease (CM– group).

### Signalment and cardiac auscultation

Of the 94 cats included, 50 (53%) were female (1 intact, 49 spayed) and 44 (47%) were male (2 intact, 42 neutered). At admission, a heart murmur was documented in 63/94 (67%), a gallop sound in 5/94 (5%), and an arrhythmia in 1/94 (1%). Sex and breed distribution, mean age and body weight, clinicopathologic findings (mean PCV, NIBP, and TT4), and the presence of a murmur are summarized in Table 2. No significant differences were observed between the CM+ and CM– groups regarding sex (Fisher’s exact test, *P* = .51), body weight (Hodges–Lehmann estimate = −0.08; 95% CI, −1.12 to 0.70; *P* = .78), or age (mean difference = −0.33; 95% CI, −2.32 to 1.67; *P* = .74).

**Table 2:** Comparison of signalment and clinicopathologic findings of cats presented for SUB placement.

	No echocardiography (*n* = 30)	Cats with cardiomyopathy (*n* = 26)	Cats without cardiomyopathy (*n* = 38)
**Mean (95% CI) age (years)**	7.41 (6.19-8.63)	9.20 (7.44-10.96)	8.87 (7.87-9.88)
**Median (IQR) body weight (kg)**	4.56 (3.42-5.58)	4.69 (3.02-5.686)	3.76 (3.36-5.38)
**Mean (95% CI) PCV (%)—admission**	31.42 (28.9-33.89)	29.96 (26.07-31.86)	28.87 (27.14-30.60)
**Mean (95% CI) PCV (%)—day of procedure**	29.16 (27.04-31.29)	26.04 (23.80-28.28)	26.37 (24.49-28.25)
**Mean (95% CI) NIBP (mmHg)**	158.89 (146.10-171.68)	149.25 (138.96-159.53)	155.32 (145.55-165.08)
**Mean (95% CI) TT4**	15.31 (10.35-20.27)	14.05 (7.93-20.17)	15.54 (4.70-26.37)
**Sex distribution (F:M)**	13:17	13:13	24:14
**Breed distribution**	Domestic shorthair (22/30), Abyssinian (1/30), Balinese (1/30), Burmese (1/30), Persian (1/30), Siamese (3/30), Tonkinese (1/30)	Domestic shorthair (24/26), Bengal (1/26), Russian blue (1/26)	Domestic shorthair (30/38), Abyssinian (1/38), Burmese (1/38), Siamese (5/38), Singapura (1/38)
**Heart murmur—admission**	8/30	24/26	31/38

Abbreviations: NIBP = noninvasive blood pressure; SUB = subcutaneous ureteral bypass; TT4 = total thyroxine.

### Echocardiographic findings

The most common indication for an echocardiogram was the presence of a murmur at admission (41/64; 64%). Other reasons included: development of a new murmur during hospitalization (3/64; 4.7%), clinical evidence of FO (12/64; 18.8%), bradycardia (1/64; 1.6%), and reassessment of preexisting hypertrophic obstructive cardiomyopathy (1/64; 1.6%). In 6 cats (9.4%), no justification was documented. Echocardiographic data and classification as mild, moderate, or severe cardiomyopathy are reported in Table 3. Mean PCV on the day of or nearest day to echocardiography was 26.9% ± 1.41%.

**Table 3:** Comparison of echocardiographic findings, duration of hospitalization, presence of anemia, development of FO, and outcomes of cats with and without cardiomyopathy.

	CM+ group (*n* = 26)	CM− group (*n* = 38)
**Echocardiographic findings**	Cardiomyopathies: 14/26—Hypertrophic (53.8%) 8/26—Hypertrophic obstructive (30.7%) 2/26—Unclassified (7.7%) 1/26—Restrictive (3.8%) 1/26—Dilated (3.8%)	12/38—Dynamic right ventricular outflow tract obstruction (31.5%) 7/38—Mild to moderate mitral regurgitation and/or tricuspid regurgitation (18.4%) 6/38—Dynamic idiopathic aortic turbulence (15.8%) 6/38—Eccentric dilation secondary to fluid administration (15.8%) 6/38—No anomaly (15.8%) 1/38—Mild aortic regurgitation (15.8%)
**Median (IQR) LA:Ao**_**SAX**_	1.34 (1.27-1.60)	1.29 (1.26-1.37)
**Severity of cardiomyopathies**	22/26—Mild (84.6%) 3/26—Moderate (11.5%) 1/26—Severe (3.8%)	NA
**Median (IQR) duration of hospitalization (days)**	4.0 (3.0-5.0)	4.0 (2.5-5.0)
**Median (IQR) survival time (days)**	345.0 (98.5-1177.0)	894.0 (0.186.0-1672.0)
**Laterality of SUB (unilateral:bilateral)**	12:14	21:17
**Cause of death**	12/17 (70.6%) renal-related 1/17 (5.9%) heart-related 2/17 (11.7%) miscellaneous 2/17 (11.7%) unknown	12/22 (54.5%) renal-related 3/22 (13.6%) heart-related 4/22 (18.2%) miscellaneous 3/22 (13.6%) unknown
**Anemia**	16/26 (61%)	27/38 (71%)
**Fluid overload**	15/26 (58%)	14/38 (37%)
**Transfusion (range per cat)**	14/26 (54%) [1-3]	20/38 (53%) [1-4]

Abbreviations: CM+ = cardiomyopathy present; CM− = cardiomyopathy absent; LA:Ao_SAX_ = left atrial-to-aortic root diameter ratio in right parasternal short axis; SUB = subcutaneous ureteral bypass.

Upon review of echocardiographic images and reports, LA:Ao_SAX_ was available in 53 of 54 cats included for statistical analysis. Two values of LA:Ao_SAX_ were not reported in the original echocardiography reports and therefore were measured retrospectively by the trained intern from recorded 2D images. Ten cats were excluded from the echocardiographic statistical analysis because imaging occurred after the onset of FO. The median LA:Ao_SAX_ was 1.34 (IQR, 1.27-1.60) in the CM+ group and 1.29 (IQR, 1.26-1.37) in the CM– group. No significant association was found between LA:Ao_SAX_ and development of FO (odds ratio [OR], 3.75; 95% CI, 0.37 to 38.0; *P* = .26) or survival time (β = 7.59; 95% CI, −9.71 to 24.89; *P* = .39). For LA:Ao_SAX_, the AUC of the ROC curve was 0.59 (95% CI, 0.46-0.71), with a sensitivity of 1.00 and a specificity of 0.20 at the optimal cut-off value of 1.18. This result was not significant (*P* = .17).

### Hospitalization and outcome

Duration of hospitalization, survival time, presence or absence of anemia, administration of a blood transfusion (including the number of transfusions per cat), and development of FO during hospitalization are summarized in Table 3.

Thirty-nine of 64 cats (61%) died or were euthanized, including 17/26 (65%) in the CM+ group and 22/38 (58%) in the CM– group. No significant differences were observed between the CM+ and CM– groups regarding duration of hospitalization (Hodges–Lehmann estimate, 0; 95% CI, −1 to 1; *P* = .54) or survival time (Hodges–Lehmann estimate, 345; 95% CI, −45 to 704; *P* = .13).

At admission, 43/64 (67%) cats had anemia, and mean PCV on the day or nearest day of echocardiography was 26.9% ± 1.41%. During hospitalization, 34/64 (53%) cats received one or more transfusions. The median number of transfusions was 1.0 (IQR, 0.0-2.0) in the CM+ group and 1.0 (IQR, 0.0-1.0) in the CM– group. Anemia was not significantly associated with survival time (Hodges–Lehmann estimate, 216 days; 95% CI, −90 to 591; *P* = .23).

Overall, 29/64 cats (45%) developed FO during hospitalization. The occurrence of FO was not significantly associated with the presence of anemia (OR, 0.78; 95% CI, 0.34-1.78; *P* = .55), number of transfusions (OR, 1.14; 95% CI, 0.71-1.84; *P* = .59), presence of cardiomyopathy (OR, 2.34; 95% CI, 0.84-6.48; *P* = .10), and unilateral or bilateral obstruction (OR, 0.65; 95% CI, 0.29-1.50; *P* = .31). No significant difference was found in the cause of death between cats with or without FO (OR, 5.00; 95% CI, 0.34-72; *P* > .24). However, the development of FO significantly impacted survival (Hodges–Lehmann estimate, 410 days; 95% CI, 95-736; *P* = .01), with longer median survival time observed in cats without FO (MST, 1020; IQR, 494-1668 days) compared to those with FO (MST, 430; IQR, 40-1260 days).

In addition, serum creatinine concentration at admission (T_0_) was significantly higher in cats that developed FO compared with those that did not, with a mean (±SD) of 11.12 mg/dL (±5.93) vs 6.16 mg/dL (±5.44), respectively (*P* < .001). The mean difference in serum creatinine concentration at admission was 5.17 (95% CI, 2.58-8.139) mg/dL. Serum creatinine concentration at the 3-month follow-up (T_3_) did not differ significantly between groups (OR, 1.00; 95% CI, 0.99-1.01; *P* = .40).

### Renal recovery

Comparison of serum creatinine concentration at admission, discharge, and 1 month and 3 months post-procedure are shown in Table 4, as well as ∆Creatinine between the different timepoints. No significant difference was found in serum creatinine concentration or ∆Creatinine between cats with or without cardiomyopathy (*P* > .30). For groups, comparisons indicated that all results at different follow-ups were significantly lower than at T_0_ (*P* < .001).

**Table 4:** Evolution and comparison of serum creatinine concentration of cats with and without cardiomyopathy.

Serum creatinine	CM+ group (*n* = 26)		CM− group (*n* = 38)		*P*-value
	Median (mg/dL)	IQR	Median (mg/dL)	IQR	
**CreaT0**	6.84	3.94-14.68	7.56	3.20-14.49	.97
**CreaT1**	2.49	1.60-3.88	2.31	1.80-3.18	.78
**CreaT2**	2.15	1.88-2.92	2.41	1.99-3.11	.33
**CreaT3**	2.68	1.72-3.53	2.22	1.88-2.65	.30
**∆Creatinine1**	−3.48	−5.89-−1.14	−2.61	−6.29-−1.09	.92
**∆Creatinine2**	−4.75	−11.79-−0.64	−2.67	−12.68-−0.85	1.00
**∆Creatinine3**	−3.90	−7.14-−0.18	−2.62	−8.56-−0.18	.51

Abbreviations: CM+ = cardiomyopathy present; CM− = cardiomyopathy absent.

A significant negative effect of serum creatinine concentration at T0 on survival was identified (β = −0.0008; 95% CI, −0.0015 to −0.0002; *P* = .01), indicating that higher serum creatinine concentrations at admission were associated with shorter survival times. However, the strength of this association was relatively weak (adjusted *R*^2^ = .05), because an increase of 1.0 mg/dL of serum creatinine concentration corresponded to a decrease of 0.08% in the probability of survival.

## Discussion

Mild cardiomyopathy is a common comorbidity in cats with benign ureteral obstruction, identified in 40% of cats treated with a SUB in our study. Notably, the presence of mild cardiomyopathy did not significantly affect duration of hospitalization, renal recovery or survival, and was not significantly associated with presumed FO. However, it remains unknown whether more severe cardiac disease would have a different impact on these outcomes. In contrast, cats with clinical suspicion of FO had significantly shorter survival time and higher serum creatinine concentrations at admission. Furthermore, admission serum creatinine concentration was negatively correlated with survival time.

Heart murmurs are detected frequently in the general population of cats, with a reported prevalence ranging from 15.5% to 40.8% in apparently healthy cats.^15^^,^^40^^,^^41^ In our study population, the frequency of heart murmurs was notably higher at 64% (41/64), and was the primary reason for requesting echocardiography. Of the cats that underwent echocardiographic evaluation, cardiomyopathy was diagnosed in 40% (26/64) of cats, with 85% (22/26) of the cats having hypertrophic cardiomyopathy (HCM) or HOCM. These findings align with previous reports identifying HCM as the most prevalent form of cardiomyopathy in cats referred for echocardiographic evaluation, accounting for approximately 60% of cases.^42^ However, the majority of cats that underwent echocardiography in our study had no structural cardiac abnormalities. This observation supports prior evidence that the presence of a heart murmur in cats is a poor predictor of underlying cardiomyopathy.^15^^,^^40^ Among non-pathologic causes of murmurs, DRVOTO was most frequently identified, accounting for 31.5% (12/38) of echocardiographic findings. Notably, 2 of these 12 cats were concurrently anemic, limiting the ability to attribute the murmur solely to DRVOTO.

In human and veterinary medicine, kidney and heart disease frequently coexist, and the interplay between both systems is complex.^43–45^ Both are generally considered risk factors for fluid retention and adverse outcomes during hospitalization, particularly in the context of IV fluid therapy and kidney disease.^30^^,^^45^^,^^46^ A decrease in cardiac output as seen in cardiac disease, whether acute or chronic, coupled with activation of the neurohormonal system, can result in a decrease in glomerular filtration rate (GFR). This alteration subsequently affects renal function, resulting in increased serum creatinine and blood urea nitrogen concentrations.^44^ Surprisingly, we failed to identify a significant association between cardiomyopathy and clinical outcomes such as FO, renal recovery, and survival. Cats with known or suspected cardiomyopathy may have received more cautious fluid management, after echocardiographic evaluation and clinical risk assessment. Echocardiography was performed in most cats with murmurs, potentially allowing for earlier identification of structural disease and more conservative fluid administration. In addition, all but 4 cats diagnosed with cardiomyopathy had normal LA size (<1.6), which may explain the lack of significant association. Therefore, our findings cannot be extrapolated to cats with advanced cardiomyopathy, in which the hemodynamic impact may be more pronounced. Only one cat in the CM+ group, diagnosed with moderate cardiomyopathy at the time of SUB placement, died after an episode of CHF 1219 days after surgery. This outcome occurred after hospitalization for vomiting, anorexia, and polydipsia in the context of chronic weight loss, during which a neoplastic process such as lymphoma was highly suspected, suggesting that the clinical decline was likely multifactorial. Close monitoring by experienced clinicians at our hospital, including serial thoracic point-of-care ultrasound (POCUS) and tailored perioperative management (eg, frequent assessment of vital signs, auscultation, and body weight measurement), may have mitigated the risk of FO in this subgroup. Therefore, the lack of effect of cardiomyopathy on outcomes in this cohort may reflect a combination of early recognition, proactive monitoring, and cautious therapeutic strategies tailored to cardiac status.

Placement of SUBs is associated with consistently good outcomes for the treatment of benign ureteral obstruction. Median survival time after surgery may vary among studies, but ranges between 762 and 923 days.^1^^,^^11^^,^^47^ Fluid overload emerged as a significant negative prognostic factor of long-term survival in our population, with affected cats demonstrating markedly shorter MST of 430 days vs 1020 days for cats with and without FO, respectively. This finding is consistent with both human and veterinary medical literature, in which FO has been associated with increased morbidity and mortality in critically ill patients.^11^^,^^26–28^^,^^30–32^ The pathophysiology of FO likely involves a combination of hemodynamic, renal, and pulmonary consequences. Excessive intravascular and interstitial fluid may exacerbate renal dysfunction by mechanisms such as increased central venous pressure, interstitial edema, and decreased GFR, contributing to a deleterious cycle of fluid retention and renal injury.^26^^,^^29^^,^^30^^,^^48^ Furthermore, FO may impair pulmonary gas exchange, decrease tissue oxygenation, and provoke systemic inflammation, all of which can contribute to worse outcomes.^22^^,^^30^^,^^48^ These findings emphasize the importance of judicious fluid therapy, particularly in cats with underlying renal or cardiovascular vulnerability.

Increased serum creatinine concentration at admission was significantly associated with both development of FO and shorter survival time. This finding likely reflects the severity of the ureteral obstruction and the extent of renal impairment at presentation. Increased serum creatinine concentration is a surrogate marker of decreased GFR and may indicate more advanced or prolonged obstruction, more nephron loss, or preexisting CKD.^8^^,^^49^ In this context, the ability of the kidneys to excrete free water and maintain fluid balance is compromised, predisposing these patients to fluid retention once IV therapy is initiated. Moreover, renal dysfunction impairs regulation of sodium and water balance, alters vascular permeability, and often is accompanied by activation of the renin–angiotensin–aldosterone system, all of which contribute to volume expansion and interstitial edema.^50^ In both human and veterinary medical studies, FO in the setting of acute kidney injury or CKD has been independently associated with adverse outcomes, including longer hospitalization, increased risk of respiratory compromise, and higher mortality.^27–31^^,^^51^ Thus, in cats with high admission serum creatinine concentration, careful fluid administration, early detection of volume status changes, and reassessment of fluid needs are critical to minimizing the risk of FO and improving prognosis.

### Limitations

Our study had several limitations inherent to its retrospective design. First, the absence of standardized diagnostic and treatment protocols across cases may have introduced variability in clinical decision-making, echocardiographic interpretation, and fluid therapy administration. Clinicians were likely to have adopted a more conservative approach to IV fluid therapy in cats diagnosed with or suspected of having cardiomyopathy, after echocardiographic evaluation and clinical risk assessment. This individualized fluid management strategy may have influenced the frequency of FO observed in this subgroup and potentially attenuated associations between cardiomyopathy and clinical outcomes.

The diagnosis of cardiomyopathy was based on echocardiographic findings, which may have been confounded by pseudohypertrophy secondary to hypovolemia at admission. Although efforts were made to account for this possibility by assessing the clinical context and reviewing repeat studies, some misclassification may have occurred. In addition, 10 cats underwent echocardiography only after the onset of FO and therefore were excluded from predictive analyses, decreasing the scope of the study.

Assessment of FO very much depends on the clinician’s experience, skill in POCUS, and interpretation of diagnostic criteria. The criteria used in our study have not been validated previously, and definitions of FO vary in the literature, which may introduce classification bias. To minimize subjectivity, FO was retrospectively diagnosed using established major and minor criteria, along with echocardiographic evaluation. However, some criteria may have been inconsistently documented or underdiagnosed in mild cases, introducing potential misclassification bias.

Furthermore, the use of sedation, variability in imaging operators, and non-standardized perioperative fluid protocols may have influenced echocardiographic measurements, classification of severity, and clinical outcomes. The absence of an objective illness severity score limited our ability to correlate illness severity with renal variables and the development of FO.

Finally, although our study included a large cohort of cats undergoing SUB placement, sample size may have been insufficient to evaluate some complications or detect small effect sizes, particularly when stratified by cardiac phenotype. A larger population of cats would allow for complex multivariate models with stronger predictive power as well as validation of the results and, in particular, those of the thresholds estimated in our study. Prospective studies using standardized imaging, validated FO definitions and criteria and detailed fluid management protocols are warranted to better clarify the relationships among cardiomyopathy, FO, and outcomes in this population. In addition, although our study focused on LA:Ao_SAX_ as an echocardiographic index, evaluation of more advanced echocardiographic parameters (eg, LV wall thickness, other markers of LA size and function, diastolic function indices, and myocardial strain) could help identify earlier predictors of FO and refine risk stratification in cats with concurrent cardiac and renal disease.

## Conclusion

Cardiac disease is a common comorbidity in cats presented for management of benign ureteral obstruction, affecting nearly half of our population. Its presence was not significantly associated with worse short- or long-term outcomes. Therefore, mild to moderate cardiomyopathy does not seem to represent a contraindication to SUB placement, although prospective studies are needed to confirm this observation, especially in more advanced forms of cardiomyopathy. Instead, FO itself emerged as a predictor of decreased survival, emphasizing the importance of early recognition and careful fluid administration. Higher serum creatinine concentration at admission also was associated with both the development of FO and worse survival, suggesting that the severity of ureteral obstruction and impaired renal function may predispose cats to volume mismanagement and worse outcomes. Our study did not identify any association between LA:Ao_SAX_ and development of FO in this population in which most cats had normal LA size. However, the findings emphasize the importance of cautious fluid administration and close monitoring, particularly in cats with azotemia or cardiovascular concerns, because fluid therapy still may be poorly tolerated in cats with more advanced cardiomyopathy and marked LA enlargement. Prospective studies using standardized imaging, including more advanced echocardiographic parameters, and treatment protocols are warranted to further investigate the complex interplay between renal and cardiac disease in this population and to guide evidence-based fluid therapy in critically ill cats.

## Off-label antimicrobial declaration

The authors declare no off-label use of antimicrobials.

## Institutional animal care and use committee or other approval declaration

The authors declare no institutional animal care and use committee or other approval was needed.

## Abbreviations

AUC: area under the curve
CHF: congestive heart failure
CHUV: Veterinary Teaching Hospital of the University of Montreal
CKD: chronic kidney disease
DRVOTO: dynamic right ventricular outflow tract obstruction
FO: fluid overload
GFR: glomerular filtration rate
HCM: hypertrophic cardiomyopathy
HOCM: hypertrophic obstructive cardiomyopathy
IBD: inflammatory bowel disease
IRIS: International Renal Interest Society
LA: left atrium
LA:AoSAX: left atrial-to-aortic root diameter ratio in right parasternal short axis
LV: left ventricle
MST: median survival time
NE: no echocardiography
NIBP: noninvasive blood pressure
OR: odds ratio
PCV: packed cell volume
POCUS: point-of-care ultrasound
ROC: receiver operating characteristic
SUB: subcutaneous ureteral bypass
TT4: total thyroxine
